# Rehabilitation approach in robot assisted total knee arthroplasty: an observational study

**DOI:** 10.1186/s12891-023-06230-2

**Published:** 2023-02-22

**Authors:** Dalila Scaturro, Fabio Vitagliani, Dario Caracappa, Sofia Tomasello, Rita Chiaramonte, Michele Vecchio, Lawrence Camarda, Giulia Letizia Mauro

**Affiliations:** 1 Department of Surgery, Oncology and Stomatology, University of Palermo, 90127 Palermo, Italy; 2 University of Catania, Via Santa Sofia 87, 95100 Catania, Italy; 3 University of Palermo, 90127 Palermo, Italy; 4 Rehabilitation Unit, AOU Policlinico Vittorio Emanuele, Via Santa Sofia 78, 95100 Catania, Italy; 5 Department of Pharmacology, Department of Biomedical and Biotechnological Sciences, University of Catania, Catania, 95124, Italy, University of Catania, Catania, Italy

**Keywords:** Knee arthroplasty, Rehabilitation, Knee osteoarthritis, NAVIO, Outcomes

## Abstract

**Background:**

The purpose of this study is to evaluate the impact of total knee arthroplasty (TKA) with the aid of Navio Robot, comparing it with standard prosthetic surgery on the functional outcomes of patients after an intensive rehabilitation program.

**Method:**

A case–control observational study was conducted on patients undergoing TKA for severe KOA. All patients underwent the same intensive hospital rehabilitation program of 14 daily sessions lasting 3 h. The following rating scales were administered: Numeric Rating Scale (NRS), Knee Society Score (KSS) and 12-Item Short Form Survey scale. Patient assessments were performed 1 week post-surgery (T0), 1 month post-surgery (T2), and 3 months post-surgery (T3). The primary outcomes were active knee extension and flexion and pain severity. The secondary outcomes were functional capacity and quality of life.

**Results:**

Using repeated measures ANOVA, we observed at T1 a statistically different difference for the treatment group compared to the control group about KSS (*p* < 0.05), pain (*p* < 0.05), and knee flexion (*p* < 0.05). No statistically significant difference between the two groups was observed for knee extension (*p* = 0.09) and the SF-12 scale (*p* = 0.52). At T2 instead, we observed a statistically significant difference for the treatment group compared to the control group as regards KSS (*p* < 0.05) and knee flexion (*p* < 0.05), while no statistically significant difference was observed for pain (*p* = 0.83), knee extension (*p* = 0.60), and the SF-12 scale (0.44).

**Conclusions:**

Our study has demonstrated that robot-NAVIO assisted knee prosthesis surgery, associated with a specific intensive rehabilitation treatment, in the short and medium term, determines good pain control, better flexion recovery and a improvement of functional capacity.

## Introduction

Knee osteoarthritis (KOA) is a chronic degenerative disease of the articular cartilage. It typically involves elderly patients, but can also affect younger people, secondary to trauma or surgery. It is characterized by pain, functional limitation and in the most severe cases it can cause deformation of the joint itself and impaired walking [1].

Total knee arthroplasty (TKA) is a definitive solution to severe KOA thanks to the medium and long-term benefits for quality of life, pain resolution and functional recovery [2].

Over the years increasingly precise surgical techniques have been developed and biocompatible and long-lasting materials have been designed. Furthermore, in the early twenty-first century, robotics was introduced in prosthetic surgery. This provides the surgeon with support in accurately making bone cuts and continuous intraoperative feedback to restore joint kinematics and soft tissue balance. Today there are several systems available, which have advantages and disadvantages. From the literature review, it emerges that there is no agreement on the improvement of implant survival by the use of robotic prosthetic surgery. It has recently been shown that the use of robotics in knee replacement is not associated with an increase in surgical time or risk of complications. It also reduces days of hospitalization and post-operative pain with a faster return to daily life and sport, as well as greater patient satisfaction [3, 4].

Furthermore has emerged a greater precision and better positioning of the implant, reducing the incidence of failures and complications [5].

Robotic systems, are designed to improve the precision of the surgical procedure by optimizing the tissue balance, restoring the joint space and the normal kinematics of the knee in prosthetic replacement operations [6].

The NAVIO™ system (Smith and Nephew, Pittsburgh, PA, USA) is a next-generation device that uses miniaturized handheld robot-assisted instrumentation that the surgeon manipulates at 6 degrees of freedom. It allows the operator to work on pre-operative planning with the support of sensors and surgical instruments connected to 3D software capable of analyzing knee movement and the three-dimensional reconstruction of the anatomy of the joint surfaces. The intervention becomes extremely personalized and minimally invasive [7].

The literature review shows the importance of early rehabilitation and a common consensus on its effectiveness after knee prosthesis, however there is no defined and shared exercise program; in fact, various rehabilitation programs have been proposed in terms of modality, duration and intensity [8].

Based on scientific evidence, optimal rehabilitation should begin immediately after surgery to reduce hospital stay and adverse events associated with hypomobility, mainly stiffness, regardless of the technique used [9].

Several publications describe the radiological and clinical results after robot-assisted surgery using NAVIO in patients undergoing unicompartmental knee replacement surgery [6, 9, 10] while few studies analyze the same related outcomes rehabilitation treatment [11].

There are no studies in the literature that have evaluated the short- and medium-term functional outcomes in patients undergoing total knee replacement surgery with surgery assisted by the NAVIO system. The purpose of the following study is to evaluate the impact of total knee replacement with the aid of Navio Robot Smith & Nephew compared to standard prosthetic surgery, on the functional outcomes of patients after an intensive rehabilitation program.

## Materials and methods

A case–control observational study was conducted at the U.O.C. of Recovery and Functional Rehabilitation of the A'O.'U. P. Paolo Giaccone of Palermo, in collaboration with the U.O.C. of Orthopedics and Traumatology of the same hospital, on patients undergoing total knee replacement surgery for severe KOA.

The study was conducted by the ethical guidelines of the Declaration of Helsinki; the local ethics committee “Palermo 1” approved the study, with the reference number 11/2021; the information and data were managed according to the guidelines of the Good Clinical Practice (GCP). All participants signed an informed consent form at the time of enrollment to collect clinical data.

Using the hospital database, we included a consecutive series of patients who underwent TKA with a Smith & Nephew NAVIO robotic system or standard surgical technique between November 2021 and November 2022.

The following inclusion criteria were used: age between 50–70 years; elective TKA surgery performed in the last 7 days for KOA grade 4 according to Kellgren-Lawrence classification; no previous knee surgery; absence of septic episodes in progress.

Exclusion criteria were: altered states of consciousness; history of inflammatory arthropathy in the same knee; bilateral knee replacement surgery; history of previous surgery in the same knee; clinically uncorrectable varus/valgus deformity ≥ 15°; ligament insufficiency (anterior cruciate ligament rupture; collateral ligament insufficiency); rheumatological pathologies; neurological movement disorders; foot, ankle, or hip disease causing significant pain or gait disturbances.

60 patients met the inclusion criteria and were included in the study. Of these, 30 patients underwent surgery with the NAVIO robot and were assigned to a treatment group, while the remaining 30 patients underwent standard surgery and were assigned to a control group.

All total knee arthroplasty procedures were performed by 2 surgeons with years of knee replacement experience, both performing a minimum of 100 TKA procedures per year. The type of knee prosthesis used was the same in all patients recruited.

Demographic information (age, gender, BMI), clinical information (prosthesis side, active ROM in extension and flexion) were taken into consideration. The following rating scales were also administered: Numeric Rating Scale (NRS) [12], for pain; Knee Society Score (KSS) [13] for functional capacity; and 12-Item Short Form Survey (SF-12) scale [14] for quality of life.

Patient assessments were performed 1 week post-surgery (T0), 1 month post-surgery (T1), and 3 months post-surgery (T2) by an expert physiatrist (D.S.).

The primary outcome measure was functional capacity, using KSS. The secondary outcomes evaluated were: active knee extension and flexion; extent of pain, using the NRS scale; and quality of life, using the SF-12 scale.

The day following our first clinical evaluation, all patients underwent the same intensive hospital rehabilitation program of 14 daily sessions lasting 3 h, under the supervision of a physiotherapist with many years of experience.

This rehabilitation program was exercise-based with the goals of improving knee ROM, regaining knee and hip muscle strength, maintaining thigh tissue length and elasticity, preventing thrombosis, and independence in activities of daily life. The rehabilitation program included a first 15-min warm-up phase using an exercise bike and treadmill. Subsequently, the following were performed: joint mobilization exercises and manual techniques for restoring knee ROM; progressive muscle-strengthening exercises targeting the ankle plantar flexors, quadriceps femoris, hamstrings, abductors, adductors, and hip flexors; balance and agility exercises; hamstring stretch exercises; stair climbing and descending exercises; and a walking program including progression with assistive devices [8, 15, 16].

### Statistical analysis

All analyzes were performed using R software (R Core Team, 2013). The sample size was calculated to detect a mean difference of KSS (0–100) between robot-assisted NAVIO surgery and standard surgery. A power analysis was conducted with the type I error set at 0.05 and the type II error at 0.15 (85% power). The estimated sample size was 35 for each group to detect the least clinically significant difference in KSS of 5.1 units. The follow-up loss was estimated to be 20%. For this reason, the number of 30 patients for each group was sufficient to prove our thesis.

For the statistical test, we used three different tests: the t-test to compare averages for quantitative variables and Mood’s median test to compare medians for ordinal variables. Instead, we used repeated measures ANOVA to test the significance of the difference between the treatment group and the control group.Results were considered statistically significant with a *P* value < 0.05.

## Results

During this study, 60 patients were recruited, 26 men (43.4%) and 34 women (56.6%), with a mean age of 66.8 ± 5.85 years and a mean BMI of 27.7 ± 3.8 kg/m2. 39 patients (65%) underwent right knee prosthesis surgery, while the remaining 21 patients (35%) underwent left knee replacement surgery. At recruitment (T0) the mean active knee flexion was 61.8 ± 20.66° and the mean active extension was 10.2 ± 8.53°. The mean pain perceived by the patients was equal to 6.71 ± 0.95 according to the NRS scale, with a mean value of the KSS equal to 38.4 ± 5.3 and of the SF-12 scale equal to 29.64 ± 4.67. No statistically significant difference was observed at baseline between the two groups examined (Table 1).

**Table 1:** General characteristics of the patients at baseline

Characteristics	Total (*n* = 60)	Treatment Group (*n* = 30)	Control Group (*n* = 30)	*p*-value
Age, mean ± SD	66,8 ± 5,85	66,2 ± 6,02	67,55 ± 5,71	0,46
Sex, n°(%)				
Male	26 (43,3)	14 (46,6)	12 (40)	0,22
Female	34 (56,7)	16 (53,4)	18 (60)	
BMI, mean ± SD (Kg/m2)	27,7 ± 3,8	27,6 ± 4,2	27,9 ± 3,2	0,79
Prosthesis Side, n°(%)				
Right	37 (61,6)	14 (46,6)	16 (53,4)	0,54
Left	23 (38,4)	16 (53,4)	14 (46,6)	
Flexion, mean ± SD (°)	61,8 ± 20,66	60,4 ± 21,6	63,33 ± 19,35	0,65
Extension, mean ± SD (°)	10,2 ± 8,53	11,05 ± 9,9	9,38 ± 6,96	0,53
NRS, mean ± SD	6,71 ± 0,95	6,9 ± 1,06	6,5 ± 0,79	0,17
KSS, mean ± SD	38,4 ± 5,3	38,7 ± 6,1	37,6 ± 5,6	0,47
SF-12, mean ± SD	29,64 ± 4,67	29,3 ± 5,01	29,95 ± 4,27	0,65

*T* Test: *p* < 0.05; Mood’s median test: *p* < 0.05

After 1 months from recruitment (T1), patients in the treatment group showed statistically significant improvements in KSS value (38.7 ± 6.1 vs 45.3 ± 4.9; < 0.05), knee flexion (60.4 ± 21.6 vs 90.2 ± 17; *p* < 0.05) and for pain (NRS: 6.9 ± 1.06 vs 5.47 ± 1.65; *p* < 0.05). No statistically significant improvement was observed for knee extension (11.05 ± 9.9 vs 9.86 ± 9.1; *p* = 0.69) and for the SF-12 scale (29.33 ± 5 0.01 vs 32.19 ± 4.98; *p* = 0.07) (Table 2). At T1 in the control group we observed a statistically significant improvement only in knee flexion (63.33 ± 19.35 vs 80.95 ± 16.57; *p* < 0.05) (Table 2).

**Table 2:** Evolution of primary and secondary outcomes at T1 and T2 in the treatment group

Characteristics	T0	T1	*p*-value	T2	*p*-value
KSS, mean ± SD	38,7 ± 6,1	45,3 ± 4,9	< 0,05	52,2 ± 5,3	< 0,05
Flexion, mean ± SD (°)	60,4 ± 21,6	90,2 ± 17	< 0,05	105,5 ± 12,6	< 0,05
Extension, mean ± SD (°)	11,05 ± 9,9	9,86 ± 9,1	0,69	4 ± 5,77	< 0,05
NRS, mean ± SD	6,9 ± 1,06	5,47 ± 1,65	< 0,05	3,52 ± 1,56	< 0,05
SF-12, mean ± SD	29,33 ± 5,01	32,19 ± 4,98	0,07	32,09 ± 2,91	< 0,05

*T* Test: *p* < 0.05

After 3 months from surgery (T2), in the treatment group we observed statistically significant improvements in the KSS (38.7 ± 6.1 vs 52.2 ± 5.3; *p* < 0.05), knee flexion (60.4 ± 21.6 vs 105.5 ± 12.6; *p* < 0.05), knee extension (11.05 ± 9.9 vs 4 ± 5.77; *p* < 0.05), pain (6.9 ± 1.06 vs 3.52 ± 1.56; *p* < 0.05) and the SF-12 scale (29.33 ± 5.01 vs 32.09 ± 2.91; *p* < 0.05) (Table 3). In the control group, statistically significant improvements were observed for KSS (37 0.6 ± 5.6 vs 48.2 ± 5.8; *p* < 0.05), for knee flexion (63.33 ± 19.35 vs 91.47 ± 14.72; p < 0.05), for knee extension (9.38 ± 6.96 vs 5.04 ± 6.93; *p* < 0.05) and for pain (6.52 ± 0.79 vs 3.43 ± 1.29; *p* < 0.05). No statistically significant improvement was instead observed for the SF-12 scale (29.95 ± 4.27 vs 31.24 ± 3.95; *p* = 0.31) (Table 3).

**Table 3:** Evolution of primary and secondary outcomes at T1 and T2 in the control group

Characteristics	T0	T1	*p*-value	T2	*p*-value
KSS, mean ± SD	37,6 ± 5,6	40,2 ± 6,1	0,09	48,2 ± 5,8	< 0,05
Flexion, mean ± SD (°)	63,33 ± 19,35	80,95 ± 16,57	< 0,05	91,47 ± 14,72	< 0,05
Extension, mean ± SD (°)	9,38 ± 6,96	7,76 ± 6,45	0,44	5,04 ± 6,93	< 0,05
NRS, mean ± SD	6,52 ± 0,79	6,1 ± 1,13	0,14	3,43 ± 1,29	< 0,05
SF-12, mean ± SD	29,95 ± 4,27	31,28 ± 3,89	0,29	31,24 ± 3,95	0,31

*T* Test: *p* < 0.05

Using repeated measures ANOVA for the analysis of the difference between the two groups, we observed at T1 a statistically different difference for the treatment group compared to the control group about KSS (*p* < 0.05), pain (*p* < 0.05), and knee flexion (*p* < 0.05). No statistically significant difference between the two groups was observed for knee extension (*p* = 0.09) and the SF-12 scale (*p* = 0.52). At T2 instead, we observed a statistically significant difference for the treatment group compared to the control group as regards KSS (*p* < 0.05) and knee flexion (*p* < 0.05), while no statistically significant difference was observed for pain ( *p* = 0.83), knee extension (*p* = 0.60), and the SF-12 scale (0.44).

## Discussion

During our study, we evaluated the impact of total knee replacement surgery with the aid of Navio Robot Smith & Nephew compared to traditional knee replacement surgery on the functional outcomes of patients. We have observed how both surgical methods give excellent functional results in patients, the surgical procedure assisted by NAVIO, compared to the traditional procedure, has resulted in statistically significant improvements in the short term as regards the improvement of ROM in flexion, pain, and ability functional of patients. Improvements in flexion ROM and functional capacity in patients remained higher than in the control group even in the short-term follow-up, however with equal efficacy on pain. The two methods, on the other hand, appeared to be superimposable in terms of improvement of ROM in knee extension and quality of life, both in the short and long term.

To date, only a few studies in the literature have compared the efficacy, in terms of clinical and functional results, of robot-assisted knee prosthetic surgery compared to the traditional technique, and most of these studies concerned unilateral knee arthroplasty [17–21].

Published studies have shown that robot-assisted surgery allows for greater precision and better implant placement [22–24].

These significant improvements in pain, knee flexion, and functional ability observed in patients undergoing robot-assisted knee arthroplasty with the NAVIO system could be attributed to the increased accuracy and ability to manipulate bone cuts to achieve the desired flexion and extension gaps. minimizing soft tissue trauma, reducing inflammatory responses, and improving soft tissue balance [25].

The results of our study agree with most of the data in the literature. The few studies conducted on the use of the NAVIO technique in patients undergoing unicompartmental knee prosthesis surgery report a better alignment of the bone heads from a radiological point of view [18–20], better outcomes to pain after operation and satisfaction of patient expectations [19] a lower revision rate [20] and shorter hospitalization time [21].

In contrast, a one- and two-year prospective study indicates that robotically assisted surgery results in improved outcomes in terms of patient well-being, and performance in sports and recreational activities, indicating that robotically assisted surgery meets or exceeds current reference standards of care for patient-reported outcomes [26].

Kayani et al. conducted a prospective cohort study comparing early functional outcomes in 40 conventional manual TKA followed by 40 robotic TKA. The authors found that robotic TKA was associated with a reduction in postoperative pain, better early maximal knee flexion at discharge, and a reduction in the incidence of postoperative stiffness [27].

Khlopas et al. conducted a prospective multicenter non-randomized study comparing 102 conventional TKA with 150 robotic TKA and found that robotic TKA was associated with greater improvements in walking and standing at 4–6 weeks and three months after surgery compared to conventional manual TKA [28].

Ren et al. recently conducted a meta-analysis of five studies with 323 robotic TKA and 251 conventional TKA and reported improvement and reported improvement in Knee Society Score (KSS) functional score and WOMAC scores in the robotic group at six months of follow-up [29].

The data present in the literature on the efficacy of robotic systems in knee arthroplasty appear rather conflicting, and the major criticalities concern the medium-long-term functional results [4, 27].

According to a meta-analysis, both total knee arthroplasty performed with a conventional technique and that involving the aid of computerized systems are reliable and safe to perform. However, the intervention performed with the robotic aid allows us to obtain better results in terms of alignment in different axes, with less blood loss, although there are no statistically significant differences in terms of clinical outcome, range of motion, and post complications. -operative [25, 30].

Kim et al. found no differences in a 10-year follow-up between subjects operated with the traditional technique and those operated with the aid of robotics in terms of overall survival, prosthesis failure, the onset of complications, and functional results [31].

According to a meta-analysis conducted by Chin et al., both robot-assisted total and unicompartmental knee arthroplasty are associated (albeit not statistically significant) with better ROM and lower revision rates. They lead to better radiological results, but without significant differences in medium- and long-term functional outcomes compared to conventional methods [32].

Finally, the prospective randomized trials conducted by Liow et al. [33] and Cho et al. [34] showed no difference between the two treatment groups concerning the Oxford Knee Score (OKS), the KSS, and the WOMAC, at the two-year and 10-year follow-up, respectively.

However, our study has some limitations, including the small sample size and the lack of long-term follow-up, which does not allow us to generalize our results. A further limitation of our study is the lack of pre-operative data regarding the patient variables examined, since they are not available within our hospital database, which could have influenced the results obtained.

Studies are needed in the future, for example, randomized clinical trials, to be able to verify the real efficacy of the robot-NAVIO-assisted total knee arthroplasty compared to the traditional method, especially as regards the long-term clinical results.

## Conclusions

Knee replacement surgery and rehabilitation are an essential combination for an optimal clinical result. Our study, though preliminary data, has demonstrated that robot-NAVIO assisted knee prosthesis surgery, associated with a specific intensive rehabilitation treatment, in the short and medium term, determines good pain control, better flexion recovery and a improvement of functional capacity. However the long-term results still remain questionable and need further studies.

## Data Availability

Data used to support the findings of this study are available from the corresponding author upon request.
